# Society Meetings

**Published:** 1896-12

**Authors:** 


### Society Meetings.

The Southern Surgical and Gynecological Association held its ninth annual meeting at Nashville, November 10, 11 and 12, 1896. It was most successful, scientifically and socially, and the attendance was large. Dr. E. S. Lewis, of New Orleans, in his presidential address, gave a review of the work of the association and spoke in high terms of the value of its annual meetings. On Tuesday evening, November 10th, a banquet was tendered the asso-tion at the Nicholson House, at which Mr. John Bell Keeble, city attorney, delivered an address of welcome that was responded to by the president.

Dr. Lewis S. McMurtry, of Louisville, chairman of the council, reported the following for membership, recommended by the council: Drs. C. P. Noble, Philadelphia; J. Munson Handley, Baltimore; E. Lomax Gwathmey, Norfolk, Va.; J. Howell Way, Waynesboro, N. C.; W. P. Matthews, Richmond, Va.; W. H. Doughty, Augusta, Ga.; W. H. Myers, Fort Wayne, Ind.; W. D. Haggard, Jr., C. R. Atchison, Paul F. Eve, J. R. Buist and C. S. Briggs, Nashville. After the association adjourned the visitors left by special train for Belle Meade to partake of a barbecue.

The following-named officers were elected for the ensuing year: President, Dr. George Ben Johnston, of Richmond, Va.; vice-presidents, Dr. F. W. McRae, of Atlanta, and Dr. W. E. Parker, of New Orleans; secretary, Dr. W. E. B. Davis, Birmingham; treasurer, Dr. A. M. Cartledge, Louisville; chairman of the committee of arrangements, Dr. H. H. Mudd, of St. Louis; member of the judicial council, Dr. E. S. Lewis. The association will hold its next meeting at St. Louis, beginning the second Tuesday in November, 1897.

---

The Buffalo Academy of Medicine held meetings during November as follows:

*Section on Surgery.*—Thursday evening, November 5th, when the following papers were read: Historical sketch of lithotomy, by Dr. John Parmenter; Comparative merits of the operations for stone in the male, by Dr. John B. Deaver, of Philadelphia; Inflammatory diseases of the prostate and its appendages, by Dr. J. Henry Dowd. Drs. Herman Mynter and William H. Heath led the discussion.

*Section on Medicine.*—Tuesday evening, November 10th, when papers were read as follows: Case of chronic diarrhea successfully treated by lavage, by Dr. S. A. Dunham; Importance of early attention to hypertrophy of the naso-pharyngeal lymphoid tissue, by Dr. F. W. Hinkel; Reflex effects in the pharynx and mouth of intranasal disease, by Dr. Horace Clark.

*Section on Pathology.*—Tuesday evening, November 17th, when the following paper was read: Pathology of appendicitis, by Dr. Herman Mynter.

*Section on Obstetrics and Gynecology.*—Tuesday evening, November 24th, when the following papers were read: Albuminuria of pregnancy: Etiology and pathology, by Dr. A. A. Jones; treatment, by Dr. Henry R. Hopkins.

---

THE Second Pan-American Medical Congress, that was held in the City of Mexico, November 16–19, 1896, appears to have been a conspicuous success. The president, Dr. Manuel Carmona y Valle, made a timely address; Dr. William Pepper spoke on the aims of the congress; and Señor Don José M. Gamboa took for his theme, International sanitary legislation. The president of the republic, General Porfiro Diaz, who attended with his cabinet, gave a brief address of welcome, expressing the pleasure of his government in being able to assist in advancing the interests of the congress. Dr. A. Vander Veer, of Albany, read a paper in the gynecological section. Among those present from New York were Dr. James D. Spencer, of Watertown, president of the medical society of the State of New York, and A. Walter Suiter, of Herkimer. The third congress is to be held at Caracas, Venezuela, in 1899.

---

THE Medical Society of the County of New York held its ninety-first annual meeting at the New York Academy of Medicine, No. 17 West Forty-third street, October 26, 1896. The attendance was large and much interest was shown in the friendly contest for offices which resulted in the election of the following: President, Landon Carter Gray; first vice-president, Robert A. Murray; second vice-president, Nathan Edwin Brill; secretary, Charles H. Avery; assistant secretary, William E. Bullard; treasurer, John S. Warren; censors, Seneca D. Powell, B. Farquhar Curtis, Floyd M. Crandall, Walter Lester Carr and Edward D. Fisher. Dr. Prince A. Morrow read a paper entitled Some practical phases of the leprosy question, and Dr. John A. Fordyce showed a number of lantern slides to illustrate remarks on diseases of the skin.

---

THE American Association of Obstetricians and Gynecologists, at its ninth annual meeting, held at Richmond, Va., elected the following-named officers for the ensuing year: President, James F. W. Ross, M. D., Toronto; vice-presidents, George Ben Johnston, M. D.,

Richmond and John C. Sexton, M. D., Rushville, Ind.; secretary, William Warren Potter, M. D., Buffalo; treasurer, Xavier O. Werder, M. D., Pittsburg. Executive council: Charles A. L. Reed, M. D., Cincinnati; Lewis S. McMurtry, M. D., Louisville; A. Vander Veer, M. D., Albany; J. Henry Carstens, M. D., Detroit; and William E. B. Davis, M. D., Birmingham. The next annual meeting was appointed to be held at the Cataract House, Niagara Falls, N. Y., Tuesday, Wednesday, Thursday and Friday, August 17, 18, 19 and 20, 1897.

---

The Western Surgical and Gynecological Association will hold its fifth annual meeting at Topeka, Monday and Tuesday, December 28 and 29, 1896. All regular physicians and surgeons are cordially invited to attend and take part in the proceedings. This is one of the prominent and successful societies in the West, and was organised some years ago at Topeka. Its meetings have all been well attended and full of interest.

---

The Southern Section of the American Laryngological, Rhinological and Otological Society will hold its next meeting at New Orleans, March 3 and 4, 1897. This date has been selected, as it will permit visiting members to see New Orleans during the Carnival Season and will enable them to secure half-rate railroad transportation.

